# Reaching Young People in Urban and Rural Communities with Mental Health and Wellbeing Support Within a Youth Sports Development Program: Integrating In-Person and Remote Modes of Service Delivery

**DOI:** 10.1007/s10578-023-01647-1

**Published:** 2024-01-13

**Authors:** Allison M. Waters, Rachel A. Sluis, Wayne Usher, Lara J. Farrell, Caroline L. Donovan, Kathryn L. Modecki, Melanie J. Zimmer-Gembeck, Mike Castle, James Hinchey

**Affiliations:** 1 School of Applied Psychology and Centre for Mental Health, Griffith University, Brisbane, QLD Australia; 2 School of Education and Professional Studies, Griffith University, Gold Coast, QLD Australia; 3 National Rugby League, Brisbane, QLD Australia

**Keywords:** Mental health, Organised sport, Youth, Technology, Intervention

## Abstract

Embedding mental health and wellbeing programs within youth sports development programs can help provide more young people with mental health support. However, delivering such programs in multiple locations across metropolitan, regional, and rural areas requires novel solutions to overcome geographic and logistical barriers. We examined the delivery of an integrated system delivered within an Australian junior rugby league program. The program included online assessment and feedback about youth mental health, as well as connection with evidence-informed resources and referral sources via parent telephone and email support. There were four methods of delivering player workshops during training sessions: (a) In-person Delivery Only, (b) In-person + Remote Real-time (video-conferenced), (c) In-person + Remote Prerecorded (video-recorded), and (d) Remote Delivery Only (video-conferenced and/or video-recorded). In-person delivered player workshops were facilitated by local rugby league personnel. Remote delivered workshops were facilitated by psychologists from the mental health research team. Participants were 671 boys (12–15 years; *M* age = 13.35; *SD* = 0.35) in 21 metropolitan, regional and rural locations. Regardless of delivery condition, players with elevated anxiety, depression and behavioural problems reported significant declines in symptoms from pre- to post-program, and those within healthy ranges did not change from pre- to post-program. Player workshop enjoyment ratings were higher in the In-person + Remote Real-time condition and the Remote Delivery Only condition than the In-person Delivery Only condition. However, non-completion of the post-program assessment across all conditions was higher than in prior studies and a comparison group of players who did not complete the program was not included. Mental health benefits may be observed across in-person and remote modes of delivering mental health workshops within youth sports programs. However, the involvement of mental health personnel, whether in-person or remotely, and mixed delivery modes, may be important for young people’s retention and satisfaction.

## Introduction

The popularity of organised community-based sport programs provides a unique opportunity to reach young people with mental health and wellbeing programs (e.g., [1, 2]). Indeed, 78.8% of young people in Australia (12–24 years of age) participate in organised sport [3]. However, logistical and ethical barriers can make it difficult to embed mental health and wellbeing programs within existing organised sport settings. For instance, service provision across vast geographical distances is difficult, tailoring to different age groups of youths is required, integrating the programs across multiple training sessions and venues must be coordinated, and there are concerns about stigma, confidentiality and conflicts of interest when organised sports personnel are involved in the mental health and wellbeing care of players (e.g., Hutchesson et al., 2021, [4, 5]).

Over five years of research on a co-designed and co-delivered youth rugby league development program for young men, we have found ways to overcome these barriers and successfully integrated a mental health and wellbeing system of care (i.e., Life-Fit-Learning) into a nationwide Australian junior rugby league program [5–8]. The system involves a three-step approach aimed at assessing young rugby league players’ mental health and wellbeing via completion of an online self-report survey (Assess step), summarising the survey information and providing immediate feedback to parents/carers rather than sports personnel to preserve player confidentiality (Reflect step), and connecting players to two types of resources: mental health and wellbeing workshops delivered within the youth sports program and the provision of mental health resources and referral options to parents/carers of those youth identified with elevated mental health symptoms (Connect step).

The Life-Fit-Learning system was developed based on core tenets of ecological systems theory [9], community-based participatory research (CBPR) frameworks [10], and implementation science frameworks [11]. Ecological systems theory draws attention to the interplay of multiple contextual levels that influence, and are influenced by, young people [9]. CBPR identifies how efforts to reach young people in the communities in which they live should ideally be done in close partnership with organisational and community members [10]. The Life-Fit-Learning system is also underpinned by core tenets of implementation science and the view that that scientific study of the process and methods of integrating research findings and evidence-based practices within settings is essential to improve the quality and effectiveness of health services and care (e.g., [12–14]).

In our prior studies with young male junior rugby league players (aged 12 to 15 years) (e.g., [5–8]), the Life-Fit Assess step was completed online by players, while the Reflect and Connect steps involved a combination of telephone and email support with parents to provide psychoeducation, access to resources and referral options for player with high levels of mental health symptoms, and four × player workshops delivered in-person [5, 7] or remotely in real-time via video-conference by psychologists on our team [8]. In a comparison with players who did not complete the program [7], the integrated system was found to be feasible to implement, highly acceptable to players, and efficacious in reducing or preventing anxiety and depression symptoms, and behavioural problems. Several other studies have demonstrated beneficial effects of mental health literacy programs delivered in-person by sports psychologists within youth sports programs on anxiety and depression literacy and resilience and reducing stigma for young men between 12 and 18 years of age (e.g., [15–18]).

However, youth sports programs are offered in urban and rural communities, often with multiple groups training simultaneously within the same session, and with varying levels of access to technology resources at training venues to provide mental health workshops remotely and in real-time. Therefore, in-person and video-conferenced delivery by mental health professionals has not always been feasible across large numbers of settings and, even if provided, the delivery is not sustainable over time. Flexible delivery models that combine both in-person and remote delivery across real-time and prerecorded modes is critical for widening access and reducing barriers to involvement.

The aim of the present study was to determine the comparative effectiveness of in-person and remote delivery of the Life-Fit system within a junior sport development program. The program involved young male rugby players in urban and rural locations across Australia. Local youth sports personnel and technology solutions were used to deliver the three steps (Assess, Reflect, Connect) of the Life-Fit system. For all participants, the Assess Step was administered via personal devices and the Reflect Step was administered via email and/or telephone calls to parents/carers by Life-Fit personnel. Importantly, the Connect step was delivered using four different multi-modal methods, while ensuring service provision across urban and rural communities. Specifically, in the Connect step, local rugby league personnel introduced the Life-Fit component of the program in-person with parents/carers during initial orientation sessions. Local rugby league wellbeing officers delivered the in-person Life-Fit player workshops and provisionally registered psychologists delivered the remote Life-Fit player workshops all under supervision form the Life-Fit team. The delivery combinations included (a) In-person Delivery Only, (b) In-person + Remote Real-time (via video-conference), (c) In-person + Remote Prerecorded (via video-recording), and (d) Remote Delivery only (video-recorded and/or video-conferenced).

Formulating hypotheses for this study involved two considerations. First, studies to date have not examined mental health outcomes and acceptability ratings from different combinations of multi-modal delivery of mental health and wellbeing interventions within youth sports programs. Second, players were rotating through on-field rugby league and strength and conditioning sessions with high levels of coach engagement and player interaction. This meant players were used to in-person activities and could have differed in levels of personal fatigue, concentration and motivation depending on when the Life-Fit workshops were completed during the session. We therefore based hypotheses on the expectation that engagement in the workshops would produce more satisfaction and more improvement in mental health, hypothesising that in-person and real-time remotely delivered Life-Fit workshops would enhance player engagement and learning and thus acceptability ratings and mental health benefits. In accord, we have observed mental health improvements and high acceptability ratings following in-person and video-conferenced delivery in separate prior studies [5, 7, 8]. Thus, we predicted that the In-person Delivery Only and In-person + Remote Real-time delivery conditions would produce stronger mental health outcomes and acceptability ratings compared to delivery modes that included only partial in-person/real-time delivery (i.e., In-person + Remote Prerecorded and Remote Delivery Only).

## Method

### Participants

A total of 671 male adolescents (12–15 years of age; *M* age = 13.35; *SD* = 0.35) enrolled in the RISE Rugby League Development Program for Australian junior rugby players across the year 2021. RISE is a multi-component program focused on rugby league skills development, strength and conditioning improvements and mental health and wellbeing to promote youth development and positive engagement in organised sport. The National Rugby League (NRL) developed the program in conjunction with Griffith University clinical and developmental psychology experts [7]. The Queensland Rugby League (QRL) in conjunction with the NRL recruited players into the program via advertising on NRL and QRL websites and information circulated to their junior sport clubs in each region prior to the season. Of the 671 players who originally enrolled, 195 players (29.06%) completed the program including both the pre- and post-RISE Assess steps, and 482 players completed the program and satisfaction ratings (71.8%). Demographic information for the 671 adolescents (by delivery condition) is provided in Table 1.

**Table 1:** Descriptive information on non-completers and completers (+ healthy range and high-risk range) as a function of delivery mode condition

	In-person + remote real-time				In-person + remote prerecorded				In-person delivery only				Remote delivery only			
Total N of Pre-RISE assessments	125				201				275				70			
*Non-completers (completed Pre-RISE but not Post-RISE assessment)*																
Percent (N)	57.6% (72)				80% (161)				69.8% (192				72.9% (51)			
Mean age (SD)	13.45 (0.87)				13.36 (0.97)				13.27 (0.96)				13.33 (0.93)			
% Born Australia (N)	80.6% (58)				95.6% (154)				94.7% (182)				96.1% (49)			
% Live parents (N)	75% (54)				72.7% (117)				81.3% (156)				90.2% (46)			
Mean anxiety (SD)	7.77 (4.70)				6.18 (4.09)				6.48 (4.38)				6.10 (3.10)			
Mean depress. (SD)	6.88 (3.80)				5.71 (3.28)				5.49 (3.90)				5.59 (3.72)			
Mean beh. prob. (SD)	2.25 (1.67)				1.86 (1.77)				1.70 (1.67)				1.53 (1.42)			
*Completers (completed Pre-RISE and Post-RISE assessments)*																
Percent (N)	42.4% (53)				20% (40)				30.2% (83)				27.1% (19)			
Mean age (SD)	13.06 (0.86)				13.41 (0.94)				13.25 (0.89)				12.89 (0.74)			
% Born Australia (N)	85.2% (46)				95% (38)				95.2% (79)				94.7% (18)			
% Live parents (N)	83.3% (45)				87.5% (35)				85.5% (71)				94.7% (18)			

	Healthy range (n = 39)		High-risk range (n = 14)		Healthy range (n = 27)		High-risk range (n = 13)		Healthy range (n = 70)		High-risk range (n = 13)		Healthy range (n = 13)		High-risk range (n = 6)	
	Pre-	Post-	Pre-	Post-	Pre-	Post-	Pre-	Post-	Pre-	Post-	Pre-	Post-	Pre-	Post-	Pre-	Post-
Mean anxiety (SD)	5.77 (3.24)	5.03 (3.18)	9.38 (5.64)	6.71 (4.63)	5.48 (3.62)	5.89 (4.85)	10.69 (6.81)	7.46 (4.58)	5.33 (3.57)	5.20 (3.93)	12.00 (5.08)	8.92 (4.33)	5.92 (3.62)	4.92 (3.98)	10.33 (7.39)	9.00 (7.69)
Mean depression (SD)	5.49 (2.87)	5.08 (3.34)	9.79 (5.19)	7.43 (5.58)	4.74 (3.05)	4.11 (3.25)	9.38 (4.09)	7.92 (5.30)	4.41 (2.98)	4.41 (3.31)	9.46 (4.41)	8.54 (4.61)	5.69 (3.43)	6.08 (4.31)	9.50 (4.51)	7.50 (2.88)
Mean beh. problems (SD)	1.33 (1.06)	1.44 (1.47)	4.29 (1.68)	2.50 (1.79)	1.48 (0.98)	1.59 (1.62)	4.00 (1.58)	2.85 (1.95)	1.24 (0.89)	1.16 (1.22)	3.69 (1.44)	2.62 (2.36)	0.92 (1.12)	1.15 (1.28)	4.00 (1.67)	2.67 (2.07)
%High-risk stabl. (N)	35.7% (5)				38.5% (5)				46.2% (6)				50% (3)			
%High-risk impr. (N)	64.3% (9)				61.5% (8)				53.8% (7)				50% (3)			

### Measures and Materials

#### Anxiety

The Revised Children’s Anxiety and Depression Scale (RCADS-25; 52; [19] Anxiety Subscale (15 items) was used to assess anxiety symptoms. A sample item is: “I worry when I think I have done poorly at something” (0 = *never*, 3 = *always*). Summing the items results in a possible range of scores from 0 to 45, with a higher score indicating a higher level of anxiety symptomology (Cronbach’s α = 0.79 at pre-assessment and α = 0.84 at post-assessment).

#### Depression

The RCADS-25 Depression Subscale (10 items; [19] was used to assess depressive symptoms. A sample item is: “I feel sad or empty” (0 = *never*, 3 = *always*). Summing the items results in a possible range of scores from 0 to 30, with a higher score indicating a higher level of depressive symptomology (Cronbach’s α = 0.76 and α = 0.77 at pre- and post-assessment, respectively).

#### Behavioural Problems

Five items from the Strengths and Difficulties Questionnaire (SDQ; 53) [20] were used to assess difficulties related to anger and externalising behaviours. A sample item is: “I get very angry and often lose my temper” (0 = *not true*, 2 = *certainly true*). Summing the items results in a possible range of scores from 0 to 10, with a higher score indicating a higher level of anger and externalising difficulties (Cronbach’s α = 0.55 and α = 0.57 at pre- and post-assessment, respectively).

#### Satisfaction Ratings

The extent to which players found the Life-Fit workshops to be helpful for improving their knowledge about mental health and wellbeing strategies and enjoyable to participate in were rated using two items sent via a weblink to their mobile phones after the final Life-Fit workshop and were rated on a five-point scale from 1 = *not at all helpful/enjoyable* to 5 = *extremely helpful/enjoyable*.

### Procedure

**Fig. 1:**
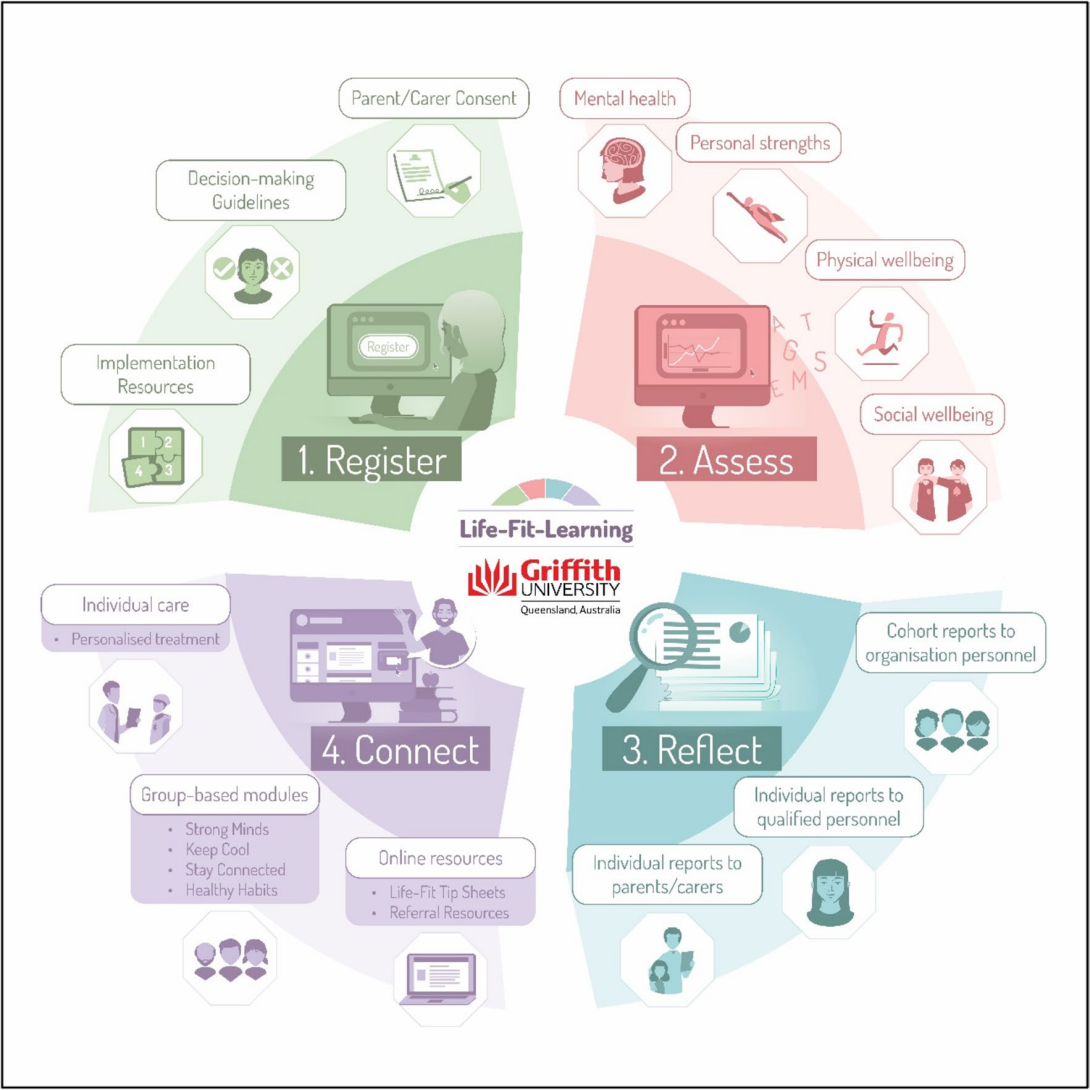
The major components of the Life-Fit-Learning system

The study had full Griffith University ethics approval (GU HREC: 2018/426). The Life-Fit-Learning System (see Fig. 1) was implemented within the rugby league development program in 17 metropolitan, regional and rural locations throughout Queensland, and four capital cities within the NRL affiliated states. Despite delays in commencing, and/or interruptions during, the delivery of the RISE program in most locations due to COVID-19 stay-at-home recommendations that were localised and intermittent in 2021, the program was successfully delivered to completion in all locations.

Participants received written informed consent from parents/carers via a link to the Life-Fit-Learning system from the RISE website. After being registered in the Life-Fit system, each participant received an email with a link to the Assess step in which they completed all measures. After submitting their responses, all participants received an email with a link to the Life-Fit-Learning website where they could download the Life-Fit Tip Sheets which provide psychoeducational resources on all topics covered within the Life-Fit system, as well as access to referral sources for further care.

Reflect Reports for each adolescent summarised scores as being in the healthy, possible risk, or probable risk range based on age- and gender-established cut-off values, normative data, or national recommendations (see [5] for details). Reports were reviewed by the first and second authors and research assistants and then emailed to parents/carers of participants.

All participants received group-based, modularised Life-Fit workshops delivered via one of four combinations depending on the geographical location, trained personnel, and technology available: (a) In-person Delivery Only, (b) In-person + Remote Real-time Delivery (video-conferenced), (c) In-person + Remote Prerecorded Delivery (video-recorded), and (d) Remote Delivery Only (video-conferenced or video-recorded). In-person workshops were delivered by one of nine QRL or NRL wellbeing officers who had completed a certificate IV in elite athlete well-being training, except in one location in which the workshops were delivered in-person and via videoconference by a provisionally registered psychologist within the Griffith Life-Fit team.

All video-conferenced workshops were delivered using Microsoft Teams by two of four Life-Fit provisionally registered psychologists located in a therapy room at the Griffith University Psychology Clinic. All video-recorded workshops were prepared by two of the same four provisionally registered psychologists using the recording function in Microsoft Teams so that power-point slide presentations and the two facilitators could be presented simultaneously during the recording. Therefore, in the In-person + Remote Real-time locations and the In-person + Remote Prerecorded locations, in-person workshops were delivered by the QRL and NRL wellbeing officers, and the video-conferenced and video-recorded workshops were delivered by Griffith University Life-Fit provisionally registered psychologists. One location was an exception in which in-person and video-conferenced sessions were delivered by the Griffith Life-Fit Team. Players in all delivery modes received the Life-Fit workshops (whether in-person or remote) within the clubhouses of the rugby league grounds at which they were participating in the RISE program in their local community. Local rugby league personnel sourced relevant technology equipment for the online workshops. This included computer and data projector facilities in a classroom style in some locations and players seated around either a desktop computer or laptop in other locations in which data projection facilities were not available.

Regardless of delivery mode, the Life-Fit workshops were between 30 and 40 min in duration and were completed by groups of players on rotation with the rugby league tactical skills component and the strength and conditioning component during each RISE session. The QRL and NRL personnel and the Griffith Life-Fit facilitators received a two-hour training workshop followed by weekly one-hour supervision via Microsoft Teams with the first and second authors and they followed a detailed Life-Fit facilitator’s manual.

The players also received a detailed Life-Fit-Learning workbook. Workshop content included four main components applied to rugby league, family, friendships and school life in each session: (1) Healthy Habits: including interactive psycho-education activities relating to healthy eating and hydration habits, bed-time and sleep routines, and safe social media usage practices, (2) Strong Minds: including interactive psycho-education activities to understand the meaning and experience of grit and optimism through rugby league-related exercises, (3) Keep Cool: including interactive exercises to practice breathing exercises, muscle relaxation and mindfulness to manage emotions, and (4) Stay Connected: including interactive psycho-education activities to learn about, and engage in, acts of kindness and gratitude (see [6–8] for further details).

After each workshop, facilitators completed a Life-Fit Workshop Session Checklist to ensure adherence to the manual and consistency across player groups. Checklists were sent to the second author within a week after the workshops and reviewed during weekly supervision. Adherence to the workshop content was high, ranging from 73 to 100% across facilitators and workshops. Players completed the satisfaction survey after the last session via a link sent to their mobile phone.

In addition to players participating in the Life-Fit workshops, parents/carers of all participants scoring in the possible or probable risk ranges for anxiety, depression and/or behavioural problems on the Reflect Reports at pre-assessment were telephoned by a member of the Griffith University Life-Fit team to provide feedback, referral options and strategies for assisting their child, regardless of the delivery modality they received. The Life-Fit Tip-Sheets were also emailed to parents/carers directly.

After completion of the RISE program, participants were again emailed a link to the Assess step, followed by numerous email reminders to complete the assessment. After completion of the post-assessment, individual Reflect reports were generated and emailed to parents/carers along with the Life-Fit-Learning Tip Sheets to facilitate access to these resources over time.

### Data Screening and Analysis

Initial analyses comparing pre-assessment variables for completers and non-completers were conducted using χ^2^ analyses and analyses of variance (ANOVA). Analyses of changes in mental health symptoms from pre- to post-RISE as a function of delivery mode condition were conducted using 4 Condition (in-person delivery only, in-person + remote real-time; in-person + remote prerecorded; remote delivery only) × 2 Risk Status (high-risk range; healthy range) × 2 Time (pre-RISE; post-RISE) mixed factorial ANOVAs. Similar to our prior studies [5–8], high-risk range participants (*n* = 46) included those who scored in the possible and probable risk ranges on the anxiety, depression and/or behavioural problems measures. Healthy range participants scored in the normative ranges on all three measures (*n* = 149). Learning and enjoyment ratings were analysed with one-way ANOVAs with 4 Condition (in-person delivery only; in-person + remote real-time; in-person + remote prerecorded; remote delivery only) as the between-subjects factor. Follow-up tests were performed using Tukey–Kramer post-hoc tests to control for the unequal sample sizes.

## Results

### Attrition Analyses

Table 1 presents descriptive information on young people who did and did not complete the post-RISE Assess step. There were no significant differences between non-completers (i.e., completers of the pre-RISE Assess step but not the post-RISE Assess step, *n* = 476) and completers (i.e., completers of both the pre- and post-RISE Assess step, *n* = 195) in participants’ age, and anxiety, depression, and behavioural problems scores (all *F*-values < 2.01, n.s.). There were also no differences in the proportion born in Australia or the proportion living at home (both χ^2^ < 1.56, n.s.). Furthermore, within each of the four defined location categories in which RISE was delivered (i.e., south-east Queensland, central Queensland, north Queensland, affiliated states), there were no significant differences in participants’ age, anxiety scores, depression scores, behavioural problems scores (all *F*-values < 1.96, n.s.), and proportion born in Australia and living at home (all χ^2^ < 0.47, n.s.) between non-completers and completers. Zi.

### Mental Health Outcomes

Mean anxiety, depression and behavioural problem scores for healthy range and high-risk range participants in each condition are presented in Table 1 and the number of high-risk range participants with each type and combination of mental health problems in each condition is presented in Table 2.

**Table 2:** Percent (and number) of participants in the high-risk range with each type or combination of mental health problems as a function of delivery mode condition

Mental health problems	In-person + remote real-time (n = 14)	In-person + remote prerecorded (n = 13)	In-person delivery only (n = 13)	Remote delivery only (n = 6)
Anxiety symptoms	7.1% (1)	15.4% (2)	15.4% (2)	16.7% (1)
Depression symptoms	21.6% (3)	7.7% (1)	15.4% (2)	16.7% (1)
Behavioural problems	50% (7)	38.5% (5)	30.7% (4)	50% (3)
Anxiety and depression symptoms	0% (0)	0% (0)	7.7% (1)	0% (0)
Anxiety and behavioural problems	7.1% (1)	0% (0)	7.7% (1)	0% (0)
Depression and behavioural problems	7.1% (1)	30.7% (4)	15.4% (2)	0% (0)
Anxiety, depression and behavioural problems	7.1% (1)	7.7% (1)	7.7% (1)	16.7% (1)

The Condition × Risk Status × Time mixed factorial ANOVAs of anxiety, depression, and behavioural problems showed similar results. For anxiety scores, there were significant main effects of Time, *F*(1, 187) = 10.64, *p* = 0.001, *np*^*2*^ = 0.05, and Risk Status, *F*(1, 187) = 26.51, *p* = 0.001, *np*^*2*^ = 0.12, and a significant Risk Status × Time interaction, *F*(1, 187) = 13.10, *p* = 0.001, *np*^*2*^ = 0.06. Thus, there were no significant condition effects or interactions involving condition, all *F*’s < 0.91. The High-Risk Range Group had significantly higher anxiety scores compared to the Healthy Range Group at pre- and post-RISE (both *p* < 0.005). In addition, follow-up analyses of the significant Risk Status × Time interaction showed that the High-Risk Range Group declined in anxiety from pre- to post-RISE (*p* < 0.001), whereas the Healthy Range Group showed no change (*p* = 0.74).

For depression scores, there were significant main effects of Time, *F*(1, 187) = 10.69, *p* = 0.001, *np*^*2*^ = 0.05, and Risk Status, *F*(1, 187) = 36.99, *p* = 0.001, *np*^*2*^ = 0.16, and a significant Risk Status × Time interaction, *F*(1, 187) = 7.24, *p* = 0.008, *np*^*2*^ = 0.04. There were no significant condition effects or interactions involving condition, all *F*’s < 0.70. The High-Risk Range Group had significantly higher depression scores compared to the Healthy Range Group at pre- and post-RISE (both *p* < 0.001). Also, the High-Risk Range Group declined in depression from pre- to post-RISE (*p* < 0.001), whereas the Healthy Range Group showed no change (*p* = 0.56).

For behavioural problem scores, there were significant main effects of Time, *F*(1, 187) = 22.32, *p* = 0.001, *np*^*2*^ = 0.11, and Risk Status, *F*(1, 187) = 90.81, *p* = 0.001, *np*^*2*^ = 0.16, and a significant Risk Status × Time interaction, *F*(1, 187) = 29.20, *p* = 0.001, *np*^*2*^ = 0.14. There were no significant condition effects or interactions involving condition, all *F*’s < 0.79. The High-Risk Range Group had significantly higher behavioural problem scores compared to the Healthy Range Group at pre- and post-RISE (both *p* < 0.001). Also, the High-Risk Range Group declined in behavioural problems from pre- to post-RISE (*p* < 0.001), whereas the Healthy Range Group showed no change (*p* = 0.52).

### Outcomes for Players Within the High-Risk Range at Pre-RISE Assessment

Overall, 22% of young people reported anxiety, depression, and/or behavioural problem scores within the high-risk ranges at the pre-RISE assessment. Of these young people, between 50 and 64% reported declines in symptoms to within the healthy range at the post-RISE assessment (see Table 1), proportions that did not differ significantly between delivery mode conditions, χ^2^ = 4.92, *p* = 0.27. To further elucidate these declines across mental health measures given high comorbidity, analyses were conducted comparing outcomes for participants who did (*n* = 22) versus did not (*n* = 19) experience symptom reductions to within the Healthy Ranges from pre- to post-RISE. The data were collapsed across delivery mode conditions given that there were no significant differences between delivery mode conditions in the omnibus analyses of symptom scores (see Table 3).

**Table 3:** Mean mental health outcome measures as a function of post-RISE outcome assessment

Measure	High-risk stable (N = 19)		High-risk improved (N = 27)	
	Pre-	Post-	Pre-	Post-
Mean anxiety (SD)	11.21 (6.10)	10.74 (5.51)	10.19 (6.37)	5.81 (3.89)
Mean depression (SD)	11.42 (4.61)	11.26 (4.82)	8.22 (3.88)	5.52 (3.18)
Mean beh. prob. (SD)	4.58 (1.61)	4.37 (1.60)	3.49 (1.39)	1.44 (1.16)
Parent/carer contactable	73.68% (14)		66.67% (18)	

As found previously, the findings of Time (pre-RISE; post-RISE) × Change Status (High-Risk Stable Group; High-Risk Improved Group) ANOVAs were similar for anxiety, depression, and behavioural problem scores. For anxiety scores, there were significant main effects of Time, *F*(1, 44) = 10.48, *p* = 0.002, *np*^*2*^ = 0.19, and Change Status, *F*(1, 44) = 4.11, *p* = 0.049, *np*^*2*^ = 0.09, and a significant Time × Change Status interaction, *F*(1, 44) = 6.78, *p* = 0.013, *np*^*2*^ = 0.13 (see Table 3). Thus, the interaction showed that anxiety scores declined significantly from pre- to post-RISE in the High-Risk Improved Group (*p* < 0.001), but not in the High-Risk Stable Group (*p* = 0.68). In addition, the two risk groups did not differ significantly at the pre-RISE assessment (*p* = 0.59), and the High-Risk Stable Group had significantly higher anxiety scores at the post-RISE assessment compared to the High-Risk Improved Group (*p* < 0.001).

For depression scores (see Table 3), there were significant main effects of Time, *F*(1, 44) = 7.55, *p* = 0.009, *np*^*2*^ = 0.15, and Change Status, *F*(1, 44) = 30.76, *p* = 0.001, *np*^*2*^ = 0.41, and a significant Time × Change Status interaction, *F*(1, 44) = 5.98, *p* = 0.019, *np*^*2*^ = 0.12. Depression scores declined significantly from pre- to post-RISE in the High-Risk Improved Group (*p* < 0.001), but not in the High-Risk Stable Group (*p* = 0.84). Notably, the High-Risk Stable Group had significantly higher depression scores at pre- (*p* = 0.014) and post-RISE (*p* < 0.001) compared to the High-Risk Improved group.

For behavioural problem scores (see Table 3), there were significant main effects of Time, *F*(1, 44) = 24.25, *p* = 0.001, *np*^2^ = 0.36, and Change Status, *F*(1, 44) = 30.76, *p* = 0.001, *np*^2^ = 0.41, and a significant Time × Change Status interaction, *F*(1, 44) = 16.37, *p* = 0.001, *np*^2^ = 0.27. Behavioural problem scores declined significantly from pre- to post-RISE in the High-Risk Improved Group (*p* < 0.001) but not in the High-Risk Stable Group (*p* = 0.69). Moreover, the High-Risk Stable Group had significantly higher behavioural problem scores at pre- (*p* = 0.032) and post-RISE (*p* < 0.001) compared to the High-Risk Improved Group. Thus, the High-Risk Stable Group had significantly higher depression symptoms and behavioural problems, but not anxiety symptoms, at the Pre-RISE assessment compared to the High-Risk Improved Group.

In terms of contact with parents/carers of players within the High-Risk Range at pre-assessment, between 66 and 73% of parents/carers of High-Risk Stable and High-Risk Improved players were contactable at the pre-RISE assessment to provide feedback and information to obtain further mental health care for their child. There were no significant differences between the High-Risk Stable and High-Risk Improved groups in the proportion of parents/carers who were versus were not contactable, χ^2^ = 2.61, *p* = 0.35 (see Table 2).

### Satisfaction Ratings

Table 4 presents the mean helpful and enjoyment ratings as a function of delivery mode condition. The one-way ANOVA of helpful ratings was not significant, *F*(3, 478) = 1.44, *p* = 0.23, *np*^2^ = 0.01. However, the one-way ANOVA of enjoyable ratings was significant, *F*(3, 478) = 3.23, *p* = 0.022, *np*^2^ = 0.02. Tukey–Kramer post-hoc tests revealed significantly higher enjoyment ratings in the In-person + Remote real-time condition and the Remote Delivery Only condition compared to the In-person Delivery Only condition (*p* = 0.045 and *p* = 0.039 respectively). No other comparisons were significant, all *p* > 0.125.

**Table 4:** Mean Life-Fit satisfaction ratings as a function of delivery-mode condition

Rating	In-person + remote real-time (n = 114)	In-person + remote prerecorded (n = 138)	In-person delivery only (n = 199)	Remote delivery only (n = 31)
Helpful	3.71 (1.12)	3.69 (1.09)	3.50 (1.16)	3.81 (0.87)
Enjoyable	3.76 (1.12)	3.63 (1.17)	3.40 (1.23)	3.90 (0.91)

## Discussion

Embedding mental health and wellbeing programs within youth sports development programs is a noteworthy opportunity for providing young people with mental health support. The current study examined varied modalities of delivering an integrated mental health and wellbeing system of care within a youth sport program to widen access and reduce barriers to program delivery. Specifically, we examined whether delivery modes involving greater in-person and real-time delivery were associated with stronger mental health outcomes and acceptability ratings relative to modalities with only partial in-person or real-time delivery. Findings indicated that online player assessment, feedback to parents/carers and player workshops resulted in significant improvements in mental health symptoms among high-risk range players, regardless of workshop delivery mode. Further, there were no significant changes in mental health symptoms among low-risk range players from pre- to post-RISE assessment across modalities. Unexpectedly, player workshops were rated as more enjoyable in the in-person + remote real-time condition and the remote delivery only condition than the in-person delivery only condition.

A key finding was that high-risk participants’ anxiety, depression and/or behavioural problem scores were found to decline significantly from pre- to post-RISE assessment, regardless of delivery mode, and corrected for unequal sample sizes. Yet, no changes were observed between delivery modes in mental health symptoms in healthy range participants from pre- to post-RISE. However, as a group, mental health symptoms among the high-risk participants remained significantly more elevated at the post-RISE assessment compared to healthy range participants. When post-RISE outcome status was considered, it was found that mental health symptoms were within the healthy range in approximately 58% of high-risk participants (regardless of delivery mode), whereas they remained significantly and stably elevated in the remaining 42% of high-risk participants. Finding that just over half of the high-risk sample improved to within the healthy range is similar to improvement rates observed in our prior studies (50–63%) [6–8]. However, depression symptoms and behavioural problem scores, but not anxiety symptom scores, were significantly higher at the pre-assessment among participants who remained in the high-risk range at post-assessment compared to those high-risk participants who improved. Future studies should consider more intensive mental health treatment, alongside participation in mental health and wellbeing programs with organised sports programs, for participants identified with more severe mental health symptoms at pre-assessment, particularly in relation to depression and behavioural problems (see [5]). Finally, the observation that mental health symptoms did not change significantly from pre- to post-RISE in healthy range participants across delivery modes also resembles findings from our prior study in which no significant changes in symptoms were observed among low-risk participants, but increased among comparison players who did not complete the RISE program [8].

Although the findings reveal the positive impact of Life-Fit within the RISE program on young rugby league players’ mental health and the lack of difference by delivery mode, the high non-completion rate of the post-RISE assessment is a limitation. Attrition and non-completion of assessments are common challenges when integrating mental health and wellbeing programs within sports development programs. For example, Vella et al. [18] included a wellbeing program as an add on to regular organised sports participation and observed that only 85 of 350 participants completed all components of the adolescent intervention including pre- and post-assessments (i.e., 75.7% non-completion rate). Thus, although the non-completion rate of 70.4% in the present study was similar to other studies, it was considerably higher than we have observed in our prior studies in which the non-completion rates ranged between 16 and 28% (Dowell et al., 2020) [7, 8]. Importantly, within the current study, there was no significant differences in completion rates between delivery modes, and there were no significant differences in mental health scores and demographic characteristics when those who did or did not complete the post-RISE Assess step were compared.

It is not known why a high proportion of young people did not complete the post-RISE survey, especially when the proportion was much higher than in our past research. However, one possibility is the pandemic and the associated delayed timing of program implementation. There were continued impacts of intermittent COVID-19 stay-at-home recommendations, workarounds to keep programs running with restrictions in place, and broader familial impacts upon players as well as parents/carers to facilitate the completion of post-RISE assessment. These disruptions along with preparation time to upscale delivery of the RISE program to statewide and capital cities meant the RISE program had a later start in the rugby league season in many locations than previous years. Recent studies of barriers and enablers to implementing mental health and wellbeing programs within sports programs identified several key contributors to successful implementation as perceived by participants, including the importance of timing of the program in the season, having adequate resources available, the inclusion of speakers with mental health credibility, and making the program engaging (Hutchesson et al., 2020) [18].

A major difference between the present study and our prior studies was the reduced direct engagement of the Griffith Life-Fit team with players and parents/carers in the present study. In our prior studies, members of the Griffith Life-Fit team provided introductory sessions on Life-Fit within RISE for parents/carers during the initial program orientation sessions, they managed the parent consent process and delivered all the Life-Fit workshops either in-person or via video-conference [6–8]. Although engaging local rugby league personnel enhanced the capacity to deliver Life-Fit within RISE statewide and across capital cities, and with a high standard of compliance with program protocols, the reduced direct contact of Life-Fit personnel with parents/carers and players may have limited their connection with the Life-Fit program, their understanding of the importance of the post-RISE assessment, and reduced mental health credibility without the involvement of mental health professionals (Hutchesson et al. 2020). Notably, the In-person + Remote Real-time condition and the Remote Delivery Only condition, in which workshops are delivered by the Griffith Life-Fit Team (whether in real-time or recorded), had significantly higher workshop enjoyment ratings than the In-person Delivery Only condition (which did not include Griffith Life-Fit facilitators) after correcting for unequal sample sizes. Another consideration is that the remote delivery conditions provide a contrast to the fully in-person rugby league skills and strength and conditioning sessions, which may increase their enjoyment. Moreover, these modes may be less likely to resemble classroom-based learning than the in-person workshops, which anecdotally, many young male adolescents have commented reminds them of school. Thus, the novelty of video-conferencing and video-recording coupled with delivery by external mental health professionals might enhance player enjoyment of mental health workshops within youth sports programs. Future studies should seek to ask participants what elements they enjoy about the workshops in addition to how much they enjoyed them.

Nevertheless, in the context of the low completions of both pre- and post-RISE assessments, the mental health findings highlight that beneficial outcomes can be observed when mental health programs are delivered in-person by local youth sports personnel with relevant background training, and remotely by psychologists, when all are supported by ongoing supervision and in the context of strong adherence to Life-Fit workshop content. However, it is noteworthy that significant logistical challenges were faced in sustaining the approach, including scheduling and availability of local sports personnel for ongoing training and supervision, high turnover and replacement of local sports personnel and their unavailability in various locations. Therefore, the findings that improvements in mental health outcomes did not vary as a function of delivery mode provides encouraging support for the combined use of local and remote delivery methods to reach young people in youth sports programs in urban and rural locations with high-quality and sustainable mental health support [7].

In addition to low completion rates at the post-assessment, there were other limitations of the present study. First, ‘high-risk’ was defined as elevated scores on anxiety, depression and/or behavioural problems. Second, although we have shown mental health and wellbeing benefits of Life-Fit within RISE in a prior comparison with players participating in the rugby league season but not the RISE program [8], the present study did not include a comparison group of participants who did not participate in RISE during the season. Thus, although the mental health outcomes are similar to our prior studies, it cannot be concluded that results were due solely to the RISE program. Moreover, as all players received the full Life-Fit system of care within the RISE program, the present study cannot determine the extent to which Life-Fit relative to other RISE components specifically contributed to outcomes and further, which components of Life-Fit contribute to mental health outcomes. Similarly, as all in-person player workshops were provided by local rugby league personnel, it cannot be ruled out that outcomes from in-person delivery would be stronger than remote delivery modes if workshops were delivered in-person by trained psychologists. Although these are interesting future directions for research, it is worth noting how difficult they would be to test within a community-based, holistic youth sports development program for all participating young people across locations in which trained psychologists are simply not available. Finally, as the study was conducted within a junior rugby league development program, it is unclear to what extent results are generalisable to other organised sports or activities.

## Summary

Taken together, the present findings suggest that an integrated mental health and wellbeing system of care (Life-Fit) for young men in a junior youth rugby league development program has positive effects on mental health (anxiety, depression, and behavioural problems) for high-risk players. Furthermore, these positive outcomes are found when this system is delivered across urban and rural settings with player workshops delivered in-person by local sports personnel and/or remotely by provisionally registered psychologists. Also, participants rated more enjoyment of the Life-Fit workshops in conditions in which some or all the sessions were remotely delivered by the Life-Fit team via video-conferencing and prerecorded workshops. Overall, the findings demonstrate how novel in-person and remote delivery combinations can efficiently and sustainably reach large numbers of young people in organised sports programs with high-quality mental health support, while maintaining the direct engagement of mental health experts to enhance player retention and satisfaction.
